# A comparison of sodium-glucose co-transporter 2 inhibitor kidney outcome trial participants with a real-world chronic kidney disease primary care population

**DOI:** 10.1093/ndt/gfae071

**Published:** 2024-03-22

**Authors:** Anna K Forbes, William Hinton, Michael D Feher, William Elson, José M Ordóñez-Mena, Mark Joy, Xuejuan Fan, Debasish Banerjee, Nicholas I Cole, Neil Munro, Martin Whyte, Rebecca J Suckling, Pauline A Swift, Simon de Lusignan

**Affiliations:** Renal Services, Epsom & St Helier University Hospitals NHS Trust, London, UK; Nuffield Department of Primary Care Health Sciences, University of Oxford, Oxford, UK; Nuffield Department of Primary Care Health Sciences, University of Oxford, Oxford, UK; Department of Clinical and Experimental Medicine, University of Surrey, Guildford, UK; Nuffield Department of Primary Care Health Sciences, University of Oxford, Oxford, UK; Nuffield Department of Primary Care Health Sciences, University of Oxford, Oxford, UK; Nuffield Department of Primary Care Health Sciences, University of Oxford, Oxford, UK; Nuffield Department of Primary Care Health Sciences, University of Oxford, Oxford, UK; Nuffield Department of Primary Care Health Sciences, University of Oxford, Oxford, UK; Renal & Transplantation Unit, St George's University Hospitals NHS Foundation Trust, London, UK; Renal Services, Epsom & St Helier University Hospitals NHS Trust, London, UK; Department of Clinical and Experimental Medicine, University of Surrey, Guildford, UK; Department of Clinical and Experimental Medicine, University of Surrey, Guildford, UK; Renal Services, Epsom & St Helier University Hospitals NHS Trust, London, UK; Renal Services, Epsom & St Helier University Hospitals NHS Trust, London, UK; Nuffield Department of Primary Care Health Sciences, University of Oxford, Oxford, UK; Royal College of General Practitioners (RCGP) Research and Surveillance Centre (RSC), Royal College of General Practitioners, London, UK

**Keywords:** cross-sectional studies, chronic kidney failure, computerized medical records systems, primary health care, sodium-glucose transporter 2 inhibitors

## Abstract

**Background:**

Observational studies suggest sodium-glucose co-transporter 2 (SGLT2) inhibitor kidney outcome trials are not representative of the broader population of people with chronic kidney disease (CKD). However, there are limited data on the generalizability to those without co-existing type 2 diabetes (T2D), and the representativeness of the Study of Heart and Kidney Protection with Empagliflozin (EMPA-KIDNEY) trial has not been adequately explored. We hypothesized that SGLT2 inhibitor kidney outcome trials are more representative of people with co-existing T2D than those without, and that EMPA-KIDNEY is more representative than previous trials.

**Methods:**

A cross-sectional analysis of adults with CKD in English primary care was conducted using the Oxford-Royal College of General Practitioners Clinical Informatics Digital Hub. The proportions that met the eligibility criteria of SGLT2 inhibitor kidney outcome trials were determined, and their characteristics described. Logistic regression analyses were performed to identify factors associated with trial eligibility.

**Results:**

Of 6 670 829 adults, 516 491 (7.7%) with CKD were identified. In the real-world CKD population, 0.9%, 2.2% and 8.0% met the Canagliflozin and Renal Events in Diabetes with Established Nephropathy Clinical Evaluation (CREDENCE), Dapagliflozin and Renal Outcomes and Cardiovascular Mortality in Patients with Chronic Kidney Disease (DAPA-CKD) and EMPA-KIDNEY eligibility criteria, respectively. All trials were more representative of people with co-existing T2D than those without T2D. Trial participants were 9–14 years younger than the real-world CKD population, and had more advanced CKD, including higher levels of albuminuria. A higher proportion of the CREDENCE (100%), DAPA-CKD (67.6%) and EMPA-KIDNEY (44.5%) trial participants had T2D compared with the real-world CKD population (32.8%). Renin–angiotensin system inhibitors were prescribed in almost all trial participants, compared with less than half of the real-world CKD population. Females were under-represented and less likely to be eligible for the trials.

**Conclusion:**

SGLT2 inhibitor kidney outcome trials represent a subgroup of people with CKD at high risk of adverse kidney events. Our study highlights the importance of complementing trials with real-world studies, exploring the effectiveness of SGLT2 inhibitors in the broader population of people with CKD.

KEY LEARNING POINTS **What was known:**

- Observational studies have indicated that sodium-glucose co-transporter 2 (SGLT2) inhibitor kidney outcome trial participants are not representative of the broader population of people with chronic kidney disease (CKD).
- Additionally, there are limited data on the generalizability to those without co-existing type 2 diabetes (T2D), and the representativeness of the EMPA-KIDNEY trial has not been adequately explored.
- No study to date has examined the generalizability of the SGLT2 inhibitor kidney outcome trials to people living with CKD in an English primary care setting.

**This study adds:**

- In this study of people with CKD in an English primary care setting, we found that <10% would have been eligible for each of the trials under investigation.
- The EMPA-KIDNEY trial was the most representative, applying to 8% of the real-world primary care CKD population. This was largely driven by the recruitment of participants without albuminuria.
- Each of the trials under investigation were more representative of people with CKD and co-existing T2D compared with those without T2D.

**Potential impact:**

- SGLT2 inhibitor kidney outcome trials represent a subgroup of people living with CKD in English primary care at high risk of adverse kidney events.
- The findings of our study highlight the importance of complementing clinical trials with real-world studies to explore the effectiveness of SGLT2 inhibitors in the broader population of people with CKD treated in real-world clinical practice.

## INTRODUCTION

Sodium-glucose co-transporter 2 (SGLT2) inhibitors are established glucose-lowering drugs for the treatment of type 2 diabetes (T2D) [1]. Randomized controlled trials demonstrated that these drugs reduce the risk of kidney failure in people with chronic kidney disease (CKD), including individuals with and without co-existing T2D [2–4]. However, these trials were conducted in participants at high risk of serious kidney events, who may not be representative of people with CKD in clinical practice. Several observational studies have explored the generalizability of trial eligibility criteria to determine the extent to which the findings apply to people with CKD in real-world clinical practice.

A study based in the USA used data from the National Health and Nutrition Examination Survey (NHANES) between 2009 and 2018 to calculate the number of adults with diabetes that met the eligibility criteria of the Canagliflozin and Renal Events in Diabetes with Established Nephropathy Clinical Evaluation (CREDENCE) trial [5]. Weighted analyses showed that 605 064 individuals with diabetes (*N* = 23 237 379) would have been eligible for CREDENCE. Similarly, a Taiwanese study found that the CREDENCE trial inclusion criteria applied to 5% of hospital patients with T2D (*N* = 1479) [6]. Another study that investigated the generalizability of the Dapagliflozin and Renal Outcomes and Cardiovascular Mortality in Patients with Chronic Kidney Disease (DAPA-CKD) trial to the US population found that the enrolment criteria were applicable to approximately 1.6 million people [7]. Meanwhile, an Italian study determined that 17% of people with CKD managed in nephrology clinics (*N* = 2887) would have been eligible for DAPA-CKD [8]. A recent US study used the NHANES data to explore the real-world applicability of the CREDENCE, DAPA-CKD, Study of Heart and Kidney Protection with Empagliflozin (EMPA-KIDNEY) and the Effect of Sotagliflozin on Cardiovascular and Renal Events in Patients with Type 2 Diabetes and Moderate Renal Impairment Who Are at Cardiovascular Risk (SCORED) trials. After applying the enrolment criteria to this cohort, almost 3 million individuals would be eligible for the EMPA-KIDNEY or SCORED trials, whilst approximately 1.6 and 0.6 million individuals would be eligible for DAPA-CKD and CREDENCE, respectively [9]. The eligible cohort from NHANES was older and had a larger proportion of females and individuals without albuminuria, compared with the EMPA-KIDNEY trial participants. Another study has determined the proportion of people with T2D that met inclusion criteria for each of three SGLT2 inhibitor kidney outcome trials (CREDENCE, DAPA-CKD and EMPA-KIDNEY) within the US population [10]. Of approximately 9 million people with T2D, between 3% and 10% met the trial inclusion criteria.

The data from these studies indicate that SGLT2 inhibitor kidney outcome trials may not be entirely representative of people with CKD. However, these studies were conducted in a limited number of different healthcare settings and countries, largely focussing on patients with CKD and co-existing T2D. Moreover, the representativeness of the EMPA-KIDNEY trial, which enrolled a more diverse range of people with CKD, including a large proportion without diabetes (54.0%) and lower levels of albuminuria (48.3% with urine albumin–creatinine ratio (ACR) <30 mg/mmol), has not been adequately explored [4]. Further analysis of large real-world populations in other healthcare settings, including people with and without co-existing T2D, is necessary to determine the generalizability to broader populations of people living with CKD. In this study, we explored the generalizability of three SGLT2 inhibitor kidney outcome trials to adults with CKD in an English primary care setting. We hypothesized that SGLT2 inhibitor kidney outcome trials are more representative of people with co-existing T2D than those without T2D, and that the EMPA-KIDNEY study is more representative of the real-world CKD population than previous trials.

The aim was to identify people in English primary care with equivalent risk of adverse kidney events to participants in the SGLT2 inhibitor kidney outcome trials.

The objectives were to:

- estimate the proportion of adults with CKD in a large nationally representative primary care population who would have been eligible for the CREDENCE, DAPA-CKD or EMPA-KIDNEY trials;
- explore reasons why individuals were ineligible;
- describe and compare the characteristics of the trial eligible CKD populations to participants enrolled in the trials; and
- explore factors associated with trial eligibility.

## MATERIALS AND METHODS

### Study design and data source

We conducted a cross-sectional analysis of adults with CKD using securely held pseudonymized data in the Primary Care Sentinel Cohort (PCSC) within the Oxford-Royal College of General Practitioners Clinical Informatics Digital Hub (ORCHID), a Trusted Research Environment. The PCSC data include computerized medical records for patients registered with primary care practices in the Oxford-Royal College of General Practitioners Research (RCGP) and Research and Surveillance Centre (RSC) network. Primary care is an ideal setting for this study because this is where most people with CKD are managed.

The Oxford-RCGP RSC is one of the oldest primary care sentinel networks in Europe, with more than 1900 volunteer practices and 19 million patients across England and Wales, which are broadly representative of the national population [11]. The PCSC is one of two subcategories of the Oxford-RCGP RSC, comprising 783 practices and 6 670 829 adults at the time of data extraction.

### Study population

We identified adults (≥18 years old) with CKD registered with primary care practices in the PCSC database on 31 December 2022. An update to a previously described ontological approach was used to identify the CKD population, using a combination of Systematized Nomenclature of Medicine (SNOMED) Clinical Terms indicating a diagnosis of CKD, estimated glomerular filtration rate (eGFR) <60 mL/min/1.73 m^2^ (based on a minimum of two serum creatinine measurements taken at least 90 days apart) and proteinuria defined as urine ACR >3 mg/mmol or urine protein creatinine ratio (PCR) >15 mg/mmol (based on a minimum of two measurements taken at least 90 days apart) [12]. eGFR was calculated using the Chronic Kidney Disease Epidemiology Collaboration (CKD-EPI) 2021 equation [13].

We identified individuals that met the eligibility criteria of three SGLT2 inhibitor kidney outcome trials, CREDENCE, DAPA-CKD and EMPA-KIDNEY. Eligibility was assessed separately for each trial and criteria were applied to the total CKD population (CKD cohort), CKD population with co-existing T2D (CKD-T2D cohort) and CKD cohort without T2D (CKD without T2D cohort). Individuals were classified as eligible if they fulfilled the published inclusion and exclusion criteria for a trial. The major eligibility criteria and how we defined them in our primary care population are summarized in Table 1. We also reported the eligibility criteria that we could not apply to our population (Supplementary data, Table S1).

**Table 1:** The major eligibility criteria of SGLT2 inhibitor kidney outcome trials and how they were defined in the primary care CKD population.

SGLT2 inhibitor kidney outcome trial eligibility criteria	Definition in primary care CKD population
CREDENCE	
Inclusion criteria	
≥30 years of age +	≥30 years of age
T2D +	Ontological approach combining SNOMED CT concepts relevant to T2D, namely diagnostic codes, blood tests results and prescriptions
HbA1c ≥6.5 and ≤12.0% +	HbA1c ≥47.5 and ≤107.7 mmol/mol
eGFR ≥30 and <90 mL/min/1.73^2^ +	eGFR ≥30 and <90 mL/min/1.73 m^2^ using CKD-EPI
Urine ACR >300 and ≤5000 mg/g +	Urine ACR >33.9 and ≤565.5 mg/mmol
On maximum tolerated dose of RAS inhibitor if not contraindicated	Current prescription for RAS inhibitor
Exclusion criteria	
NYHA Class IV congestive heart failure	Coding of NYHA Class IV congestive heart failure
Known significant liver disease	Coding of liver cirrhosis
Maintenance dialysis	Ontological approach combining SNOMED CT concepts relevant to receiving dialysis in the context of ESKD
Kidney transplantation	Ontological approach combining SNOMED CT concepts relevant to having a kidney transplant
T1D	Ontological approach combining SNOMED CT concepts relevant to T1D, namely diagnostic codes, blood tests results and prescriptions
Receiving combined ACE inhibitor and ARB treatment	Current prescription for both ACE inhibitor and ARB
DAPA-CKD	
Inclusion criteria	
≥18 years of age +	≥18 years of age
eGFR ≥25 and ≤75 mL/min/1.73 m^2^ +	eGFR ≥25 and ≤75 mL/min/1.73 m^2^ using CKD-EPI
Urine ACR ≥200 and ≤5000 mg/g +	Urine ACR ≥22.6 and ≤565 mg/mmol
On maximum tolerated dose of RAS inhibitor if not contraindicated	Current prescription for RAS inhibitor
Exclusion criteria	
Autosomal dominant polycystic kidney disease	Coding of autosomal dominant polycystic kidney disease
Autosomal recessive polycystic kidney disease	Coding of autosomal recessive polycystic kidney disease
Lupus nephritis	Coding of lupus nephritis
ANCA-associated vasculitis	Coding of ANCA-associated vasculitis
History of organ transplantation	Ontological approach combining SNOMED CT concepts relevant to having an organ transplant
T1D	Ontological approach combining SNOMED CT concepts relevant to type 1 diabetes, namely diagnostic codes, blood tests results and prescriptions
NYHA Class IV congestive heart failure	Coding of NYHA Class IV congestive heart failure
EMPA-KIDNEY	
Inclusion criteria	
≥18 years of age +	≥ 18 years of age
On maximum tolerated dose of RAS inhibitor + one of the following 2 groups:	Current prescription for RAS inhibitor
eGFR ≥20 and <45 mL/min/1.73 m^2^ or	eGFR ≥20 and <45 mL/min/1.73 m^2^ using CKD-EPI
eGFR ≥45 and <90 mL/min/1.73 m^2^ + urine ACR ≥200 mg/g (or urine PCR ≥300 mg/g)	eGFR ≥45 and <90 mL/min/1.73 m^2^ using CKD-EPI + urine ACR ≥22.6 mg/mmol (or urine PCR ≥33.9 mg/mmol)
Exclusion criteria	
T2D + prior atherosclerotic CVD (defined as IHD, stroke, PAD) + eGFR >60 mL/min/1.73 m^2^	Ontological approach combining SNOMED CT concepts relevant to T2D + coding of atherosclerotic CVD + eGFR >60 using CKD-EPI
Autosomal dominant polycystic kidney disease	Coding of autosomal dominant polycystic kidney disease
Autosomal recessive polycystic kidney disease	Coding of autosomal recessive polycystic kidney disease
Maintenance dialysis	Ontological approach combining SNOMED CT concepts relevant to receiving dialysis in the context of ESKD
Kidney transplantation	Ontological approach combining SNOMED CT concepts relevant to having a kidney transplant
T1D	Ontological approach combining SNOMED CT concepts relevant to type 1 diabetes, namely diagnostic codes, blood tests results and prescriptions
Receiving combined ACE inhibitor and ARB treatment	Current prescription for both ACE inhibitor and ARB

For each criterion we identified the nearest match from routine primary care data, using a combination of demographics, diagnostic tests, prescriptions and variables curated from SNOMED CT using our ontological approach. All laboratory measurements were based on the most recently recorded values prior to 31 December 2022.

ACE inhibitor, angiotensin-converting enzyme inhibitor; ANCA-associated vasculitis, antineutrophilic cytoplasmic antibody associated vasculitis; ARB, angiotensin receptor blocker; ESKD, end-stage kidney disease; HbA1c, glycated haemoglobin; IHD, ischaemic heart disease; NYHA, New York Heart Association; PAD, peripheral arterial disease; SNOMED CT, SNOMED Clinical Terms.

### Data preparation

We extracted demographic and clinical characteristics of the CKD population including clinical measures, comorbidities and prescribed medications. Data were captured at the time of extraction using the most recently available information prior to the 31 December 2022. A current prescription was defined as a prescription for a drug within that class within the last 90 days.

Ethnicity was grouped into five categories (White, Asian, Black, Mixed, Other), based on the Office for National Statistics definitions [14]. Socioeconomic status was determined by the Index of Multiple Deprivation (IMD) score, which was converted into quintiles ranging from 1 (most deprived) to 5 (least deprived) [15]. IMD score was based on the postcode of the individual's registered home address. Continuous data were cleaned, and outlying values excluded and assigned as missing based on expert opinion within the study team, and previously published ranges (Supplementary Appendix) [16].

Information about the characteristics of trial participants were extracted from published data [2–4].

### Missing data

We reported missing data when describing the characteristics of the CKD population and addressed missing data to investigate factors associated with trial eligibility. We assumed missing data for ethnicity were unlikely to be missing at random. Individuals with missing ethnicity data were assigned to the ‘White’ ethnicity category. Practice postcode was used to infer socioeconomic status where data were missing.

Clinical measures recorded ≥2 years prior to the 31 December 2022 were assigned as missing. We assumed that missing data for clinical measures were missing at random, and that any systematic differences between missing values and observed values could explained by differences in the observed data [17]. Multivariate Imputation by Chained Equations was used to impute missing values [18]. We made multiple predictions (*N* = 5) for each missing value, creating multiple ‘complete’ datasets which were combined using Rubin's rules.

### Outcome measures

The primary outcome was the proportion of the CKD population who would have been eligible for the CREDENCE, DAPA-CKD or EMPA-KIDNEY trials according to the enrolment criteria.

The secondary outcomes were to describe the characteristics of the trial eligible CKD populations and compare them to participants enrolled in the SGLT2 inhibitor kidney outcome trials, and explore factors associated with trial eligibility.

### Statistical analysis

Descriptive statistics were used to report the primary outcome and describe the characteristics of the CREDENCE, DAPA-CKD and EMPA-KIDNEY trial eligible CKD populations. Means (standard deviation) or medians [interquartile range (IQR)] were used to describe continuous variables, and frequencies and percentages were used to describe categorical variables.

The primary outcome was calculated separately for each trial by dividing the number of patients in each population that fulfilled the key eligibility criteria by the total population (CKD cohort, CKD-T2D cohort and CKD without T2D cohort). If an individual was missing data for clinical measures relating to the eligibility criteria (e.g. eGFR or urine ACR), we assumed that they did not meet the eligibility criteria. We also reported the proportion of individuals excluded by each eligibility criteria, separately for each trial.

The secondary outcomes were reported separately for each trial. We selected characteristics based on those reported in the clinical trials, including demographics, clinical measures, comorbidities and prescribed medications.

We compared the characteristics of the trial eligible CKD cohorts with those included in the trials using standardized differences (st. diff.) between means or proportions [19]. Meaningful differences between values were set at >0.1. Data required for this analysis were extracted from information reported in the intervention arms of the clinical trials.

Logistic regression models were created to investigate factors associated with trial eligibility and to determine the phenotype of patients eligible for each trial. We constructed separate models for CREDENCE, DAPA-CKD and EMPA-KIDNEY eligibility. Variables included in the models were pre-specified as age (years), sex (male, female), ethnicity (White, Asian, Black, Mixed, Other), IMD quintile (1–5), Cambridge Multi Morbidity Score (CMMS) [20], history of T2D, heart failure or cardiovascular disease (CVD) (absent, present) and current prescription for a diuretic or statin (absent, present). Odds ratios (OR) with 95% confidence intervals (CI) and *P*-values were reported for each variable.

All data analyses were undertaken in R version 4.3.0 (2023-04-21).

### Sensitivity analyses

We performed a complete case analysis for the primary outcome, which we defined as individuals who had both an eGFR and urine ACR recorded within 2 years of 31 December 2022. Using the complete cases only CKD population, we then recalculated the proportion of CKD patients that met trial eligibility criteria for each trial.

We performed two sensitivity analyses of the logistic regression models, exploring factors associated with trial eligibility using complete cases, to check consistency of the findings with those of the primary analysis. For the first sensitivity analysis, we defined complete cases as individuals with CKD who had recorded measurements for all variables in the logistic regression model. For the second sensitivity analysis, we further defined this to include only those in the first sensitivity analysis who had both an eGFR and urine ACR recorded within 2 years of the 31 December 2022.

### Ethical approval

Ethical approval for the study was granted by the Medical Sciences Interdivisional Research Ethics Committee, University of Oxford in December 2021 (reference number: R78841/RE001) and the St George's Research Ethics Committee, Joint Research and Enterprise Services, St George's University of London in January 2022 (reference number: 2022.0002).

## RESULTS

### Representativeness results

**Figure 1:**
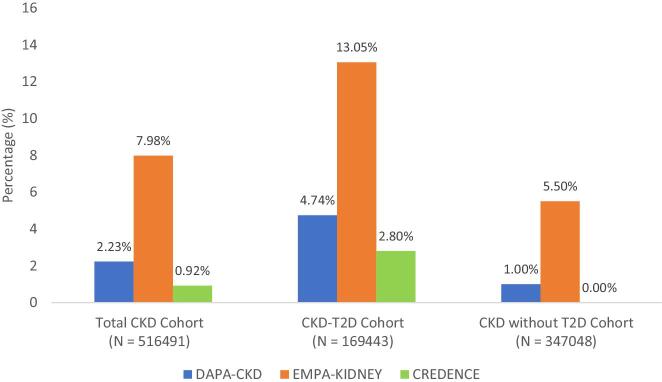
Proportion of patients eligible for each SGLT2 inhibitor kidney outcome trial for the total CKD cohort and stratified by T2D status; primary analysis. The blue represents the CKD population eligible for the DAPA-CKD study. The red represents the CKD population eligible for the EMPA-KIDNEY study. The green represents the CKD population eligible for the CREDENCE study. *N* refers to the total number of people in the cohort.

Of 6 670 829 adults, we identified 516 491 (7.7%) with CKD, including 32.8% (*n* = 169 443) with co-existing T2D. In the real-world CKD population, 0.9% (*n* = 4740), 2.2% (*n* = 11 516) and 8.0% (*n* = 41 209) met the CREDENCE, DAPA-CKD and EMPA-KIDNEY eligibility criteria, respectively (Fig. 1).

CREDENCE eligibility criteria applied to 2.8% (*n* = 4740) of the CKD-T2D population, but the eligibility criteria did not include the CKD population without T2D. The DAPA-CKD eligibility criteria applied to 4.7% (*n* = 8036) of the CKD-T2D population and 1.0% (*n* = 3480) of the CKD without T2D population, whilst the EMPA-KIDNEY eligibility criteria applied to 13.1% (*n* = 22 114) of the CKD-T2D population and 5.5% (*n* = 19 095) of the CKD without T2D population.

There were multiple reasons why individuals did not meet trial eligibility criteria (Supplementary data, Figs S2 and S3). Of those who were ineligible for the CREDENCE (*n* = 164 703), DAPA-CKD (*n* = 504 975) and EMPA-KIDNEY (*n* = 475 282) trials, over one-third were not prescribed a renin–angiotensin system (RAS) inhibitor [37.8% (*n* = 62 183), 56.1% (*n* = 283 316) and 59.6% (*n* = 283 316), respectively].

Of the CKD-T2D cohort ineligible for CREDENCE due to not being prescribed a RAS inhibitor (*N* = 62 183), 83.8% (*n* = 52 135) did not meet albuminuria criteria and 7.9% (*n* = 4937) did not have an assessment of albuminuria. Of those ineligible for DAPA-CKD due to not being prescribed a RAS inhibitor (*N* = 283 316), 55.8% (*n* = 158 152) did not meet albuminuria criteria and 39.8% (*n* = 112 751) did not have an assessment of albuminuria. Of those ineligible for EMPA-KIDNEY due to not being prescribed a RAS inhibitor (*N* = 283 316), 41.7% (*n* = 118 171) did not meet albuminuria criteria and 17.7% (*n* = 50 198) did not have an assessment of albuminuria.

Of the CREDENCE ineligible CKD-T2D population, 87.5% (*n* = 144 134) had either no albuminuria or albuminuria below the threshold (defined as urine ACR ≤33.9 mg/mmol), whilst 66.3% (*n* = 334 742) of the DAPA-CKD and 50.9% (*n* = 241 708) of the EMPA-KIDNEY ineligible total CKD populations had either no albuminuria or albuminuria below the threshold (DAPA-CKD; urine ACR <22.6 mg/mmol, EMPA-KIDNEY; urine ACR <22.6 mg/mmol in those with an eGFR ≥45 and <90 mL/min/1.73 m^2^). Absence of albuminuria assessment was an issue in 5.5% (*n* = 9061) of the CREDENCE ineligible cohort, 29.2% (*n* = 147 466) of the DAPA-CKD ineligible cohort and 15.6% (*n* = 74 353) of the EMPA-KIDNEY ineligible cohort. Less than 10% of those who were ineligible had one of the exclusion criteria [CREDENCE; 3.5% (*n* = 5696), DAPA-CKD; 2.3% (11 482) and EMPA-KIDNEY; 8.4% (39 776)].

### Comparison of characteristics

Trial participants were younger compared with the trial eligible primary care CKD populations (Tables 2 and 3). The EMPA-KIDNEY trial had a lower proportion of females compared with the trial eligible population (33.2% females vs 49.0% females, st. diff. = 0.33), whilst the proportion of females was similar in the DAPA-CKD and CREDENCE trials when compared with their respective trial eligible populations (DAPA-CKD; 32.9% females vs 34.0% females, st. diff. = 0.02, CREDENCE; 34.6% females vs 32.7% females, st. diff. = 0.04). DAPA-CKD and EMPA-KIDNEY participants had lower burden of CVD and heart failure compared with their respective trial eligible populations (DAPA-CKD: 37.8% CVD vs 54.7% CVD, st. diff. = 0.34, 10.9% heart failure vs 18.6% heart failure, st. diff. = 0.22; EMPA-KIDNEY: 26.1% CVD vs 48.0% CVD, st. diff. = 0.47, 9.8% heart failure vs 23.7% heart failure, st. diff. = 0.38), whilst a higher proportion of CREDENCE participants had CVD compared with the trial eligible CREDENCE population (50.5% CVD vs 37.8% CVD, st. diff. = 0.26). In all three trials, participants had a lower eGFR (CREDENCE: 56.3 ± 18.2 mL/min/1.73 m^2^ vs 58.7 ± 16.7 mL/min/1.73 m^2^, st. diff. = 0.14; DAPA-CKD: 43.2 ± 12.3 mL/min/1.73 m^2^ vs 50.6 ± 13.6 mL/min/1.73 m^2^, st. diff. = 0.57; EMPA-KIDNEY: 37.4 ± 14.5 mL/min/1.73 m^2^ vs 44.1 ± 14.4 mL/min/1.73 m^2^, st. diff. = 0.46) and more albuminuria [CREDENCE: 104.3 mg/mmol (IQR 51.9–202.7 mg/mmol) vs 68.5 mg/mmol (IQR 46.7–119.9 mg/mmol); DAPA-CKD: 109.1 mg/mmol (IQR 53.3–215.0 mg/mmol) vs 51.6 mg/mmol (IQR 32.7–98.1 mg/mmol); EMPA-KIDNEY: 37.4 mg/mmol (IQR 5.2–119.9 mg/mmol) vs 5.9 mg/mmol (IQR 1.5–34.3 mg/mmol)] than the trial eligible populations.

**Table 2:** Characteristics of the trial eligible CKD cohorts and participants enrolled into the DAPA-CKD and EMPA-KIDNEY trials.

	DAPA-CKD trial			EMPA-KIDNEY trial			**CKD cohort**		
Characteristic	Trial cohort (*N* = 2152)	Trial eligible CKD cohort (*N* = 11 516)	st. diff.	Trial cohort (*N* = 3304)	Trial eligible CKD cohort (*N* = 41 209)	st. diff.	**Total CKD cohort (*N* = 516 491)**	**st. diff.^a^**	**st. diff.^b^**
Age, years	61.8 ± 12.1	73.1 ± 12.7	0.91	63.9 ± 13.9	78.0 ± 11.7	1.10	70.7 ± 16.9	0.61	0.44
Female sex, *n* (%)	709 (32.9)	3910 (34.0)	0.02	1097 (33.2)	20 208 (49.0)	0.33	279 730 (54.2)	0.44	0.43
Ethnicity, *n* (%)									
White	1124 (52.2)	9088 (78.9)	0.59	1939 (58.7)	35 032 (85.0)	0.61	437 504 (84.7)	0.75	0.60
Asian	749 (34.8)	1213 (10.5)	0.61	1194 (36.1)	2461 (6.0)	0.79	27 021 (5.2)	0.80	0.83
Black	104 (4.8)	562 (4.9)	0.00	128 (3.9)	1341 (3.3)	0.03	16 724 (3.2)	0.08	0.04
Mixed		125 (1.1)		14 (0.4)	261 (0.6)	0.03	4232 (0.8)		0.05
Other	175 (8.1)	106 (0.9)	0.35	29 (0.9)	246 (0.6)	0.03	3529 (0.7)	0.37	0.02
Current smoker, *n* (%)	283 (13.2)	1148 (10.0)	0.10		2662 (6.5)		35 690 (6.9)	0.21	
Comorbidities, *n* (%)									
T1D				34 (1.0)			3802 (0.7)		0.03
T2D	1455 (67.6)	8036 (69.8)	0.05	1470 (44.5)	22 114 (53.7)	0.18	169 443 (32.8)	0.74	0.24
Cardiovascular disease (DAPA-CKD definition)	813 (37.8)	6304 (54.7)	0.34				208 195 (40.3)	0.05	
Cardiovascular disease (EMPA-KIDNEY definition)				861 (26.1)	19 800 (48.0)	0.47	167 627 (32.5)		0.14
Heart failure	235 (10.9)	2143 (18.6)	0.22	324 (9.8)	9777 (23.7)	0.38	58 276 (11.3)	0.01	0.05
Hypertension		10 606 (92.1)			37 452 (90.9)		349 096 (67.6)		
Blood pressure, mmHg									
Systolic	136.7 ± 17.5	138.9 ± 18.6	0.12	136.4 ± 18.1	135.6 ± 17.9	0.04	133.5 (16.8)	0.19	0.17
Diastolic	77.5 ± 10.7	75.7 ± 11.6	0.16	78.1 ± 11.7	73.6 ± 11.2	0.39	75.8 (10.8)	0.16	0.20
Body mass index, kg/m^2^	29.4 ± 6.0	29.9 ± 6.3	0.08	29.7 ± 6.7	29.4 ± 6.2	0.05	28.5 (6.3)	0.15	0.18
Weight, kg	81.5 ± 20.1	84.9 ± 20.4	0.17		81.1 ± (19.3)		79.1 (19.6)	0.12	
eGFR									
Mean, mL/min/1.73 m^2^	43.2 ± 12.3	50.6 ± 13.6	0.57	37.4 ± 14.5	44.1 ± 14.4	0.46	68.4 ± 23.2	1.36	1.60
Distribution (DAPA-CKD categories), *n* (%)									
<30 mL/min/1.73 m^2^	293 (13.6)	861 (7.5)	0.20				15 103 (2.9)	0.40	
≥30–<45 mL/min/1.73 m^2^	979 (45.5)	3402 (29.5)	0.34				48 706 (9.4)	0.88	
≥45–<60 mL/min/1.73 m^2^	646 (30.0)	3960 (34.4)	0.09				139 403 (27.0)	0.07	
≥60 mL/min/1.73 m^2^	234 (10.9)	3293 (28.6)	0.46				290 952 (56.3)	1.10	
Distribution (EMPA-KIDNEY categories), *n* (%)									
<30 mL/min/1.73 m^2^				1131 (34.2)	4326 (10.5)	0.59	15 103 (2.9)		0.88
≥30–<45 mL/min/1.73 m^2^				1467 (44.4)	26 378 (64.0)	0.40	48 706 (9.4)		0.86
≥45 mL/min/1.73 m^2^				706 (21.4)	10 505 (25.5)	0.10	430 355 (83.3)		1.58
Urine ACR									
Median (IQR), mg/mmol	109.1 (53.3–215.0	51.6 (32.7–98.1)		37.4 (5.2–119.9)	5.9 (1.5–34.3)		1.9 (0.8–5.5)		
Distribution (DAPA-CKD categories), *n* (%)									
>113 mg/mmol	1048 (48.7)	2410 (20.9)	0.61				7874 (1.5)	1.30	
Distribution (EMPA-KIDNEY categories), *n* (%)									
<3 mg/mmol				665 (20.1)	15 021 (36.5)	0.37	227 057 (44.0)		0.53
≥3 to ≤30 mg/mmol				927 (28.1)	12 562 (30.5)	0.05	114 628 (22.2)		0.14
>30 mg/mmol				1712 (51.8)	10 631 (25.8)	0.55	27 340 (5.3)		1.20
Medications, *n* (%)									
RAS inhibitor	2117 (98.4)	11 516 (100)	0.18	2831 (85.7)	41 209 (100)	0.58	233 175 (45.1)	1.47	0.94
ACE inhibitor	673 (31.3)	7060 (61.3)	0.63		24 426 (59.3)		146 789 (28.4)	0.06	
ARB	1444 (67.1)	4561 (39.6)	0.57		16 783 (40.7)		87 641 (17.0)	1.18	
Diuretic	928 (43.1)	4169 (36.2)	0.14	1362 (41.2)	17 844 (43.3)	0.04	117 887 (22.8)	0.44	0.40
Statin	1395 (64.8)	8668 (75.3)	0.23	2190 (66.3)	28 837 (70.0)	0.08	256 396 (49.6)	0.31	0.34
SGLT2 inhibitor	2152 (100)	2063 (17.9)	3.03	3304 (100)	4745 (11.5)	3.92	29 718 (5.8)	5.70	5.70

The ‘±’ values are means ± standard deviations. Percentages may not total 100% due to rounding.

^a^Denotes comparison of the total CKD cohort with patients enrolled in the DAPA-CKD trial.

^b^Denotes comparison of the total CKD cohort with patients enrolled in the EMPA-KIDNEY trial.

We defined CVD according to the definitions used in the DAPA-CKD and EMPA-KIDNEY studies. In DAPA-CKD it included a history of peripheral artery disease, angina pectoris, myocardial infarction, percutaneous coronary intervention, coronary-artery bypass grafting, heart failure, valvular heart disease, abdominal aorta aneurysm, atrial fibrillation, atrial flutter, ischaemic stroke, transient ischaemic attack and haemorrhagic stroke. In EMPA-KIDNEY it included a history of myocardial infarction, heart failure, stroke, transient ischaemic attack or peripheral arterial disease.

ACE inhibitor, angiotensin-converting enzyme inhibitor; ARB, angiotensin receptor blocker; HbA1c, glycated haemoglobin.

**Table 3:** Characteristics of the trial eligible CKD-T2D cohort and participants enrolled into the CREDENCE trial.

	CREDENCE trial				
Characteristic	Trial cohort (*N* = 2202)	Trial eligible CKD-T2D cohort (*N* = 4740)	st. diff.^a^	Total CKD-T2D cohort (*N* = 169 443)	st. diff.^b^
Age, years	62.9 ± 9.2	72.4 ± 11.2	0.93	73.1 ± 12.9	0.91
Female sex, *n* (%)	762 (34.6)	1548 (32.7)	0.04	79 237 (46.8)	0.25
Ethnicity, *n* (%)					
White	1487 (67.5)	3502 (73.9)	0.14	136 377 (80.5)	0.30
Asian	425 (19.3)	700 (14.8)	0.12	16 196 (9.6)	0.28
Black	112 (5.1)	255 (5.4)	0.01	7428 (4.4)	0.03
Other	178 (8.1)	47 (1.0)	0.35	1486 (0.9)	0.03
Current smoker, *n* (%)	341 (15.5)	521 (11.0)	0.13	14 646 (8.6)	0.21
Comorbidities, *n* (%)					
Cardiovascular disease (CREDENCE definition)	1113 (50.5)	1791 (37.8)	0.26	54 647 (32.3)	0.38
Heart failure	329 (14.9)	821 (17.3)	0.07	28 111 (16.6)	0.05
Hypertension	2131 (96.8)	4369 (92.2)	0.20	138 028 (81.5)	0.51
Blood pressure, mmHg					
Systolic	139.8 ± 15.6	140.5 ± 18.5	0.04	134.3 ± 17.2	0.33
Diastolic	78.2 ± 9.4	75.6 ± 11.5	0.25	74.9 ± 10.8	0.33
Body mass index, kg/m^2^	31.4 ± 6.2	31.0 ± 6.6	0.06	30.3 ± 6.6	0.17
HbA1c, mmol/mol	67.2 ± 14.2	64.9 ± 13.8	0.16	56.7 ± 17.1	0.67
eGFR					
Mean, mL/min/1.73 m^2^	56.3 ± 18.2	58.7 ± 16.7	0.14	68.2 ± 24.1	0.56
Distribution (CREDENCE categories), *n* (%)					
<15 mL/min/1.73 m^2^	1 (0.0)	0 (0.0)	0.03	1408 (0.8)	0.12
≥15–<30 mL/min/1.73 m^2^	83 (3.8)	0 (0.0)	0.28	5821 (3.4)	0.02
≥30–<45 mL/min/1.73 m^2^	594 (27)	1219 (25.7)	0.03	20 613 (12.2)	0.38
≥45–<60 mL/min/1.73 m^2^	630 (28.6)	1357 (28.6)	0.00	42 521 (25.1)	0.08
≥60–<90 mL/min/1.73 m^2^	788 (35.8)	2164 (45.7)	0.20	61 271 (36.2)	0.01
≥90 mL/min/1.73 m^2^	105 (4.8)	0 (0)	0.32	37 140 (21.9)	0.52
Urine ACR					
Median (IQR), mg/mmol	104.3 (51.9–202.7)	68.5 (46.7–119.9)		3.3 (1.2–10.1)	
Distribution (CREDENCE categories), *n* (%)					
<3 mg/mmol	16 (0.7)	0 (0)	0.12	74 034 (43.7)	1.21
≥3–≤30 mg/mmol	251 (11.4)	0 (0)	0.51	68 406 (40.4)	0.70
>30–≤300 mg/mmol	1702 (77.3)	4508 (95.1)	0.53	16 684 (9.8)	1.86
>300 mg/mmol	233 (10.6)	232 (4.9)	0.21	1258 (0.7)	0.44
Medications, *n* (%)					
RAS inhibitor	2201 (100)	4740 (100)	0.03	107 260 (63.3)	1.07
Diuretic	1026 (46.6)	1764 (37.2)	0.19	53 000 (31.3)	0.32
Statin	1538 (69.8)	4043 (85.3)	0.38	121 355 (71.6)	0.04
SGLT2 inhibitor	2202 (100)	1261 (26.6)	2.35	29 705 (17.5)	3.07

The ‘±’ values are means ± standard deviations. Percentages may not total 100% due to rounding.

^a^Denotes comparison of the trial eligible CKD-T2D cohort with patients enrolled in the CREDENCE trial.

^b^Denotes comparison of the total CKD-T2D cohort with patients enrolled in the CREDENCE trial.

We defined CVD according to the definitions used in the CANVAS study and it included a history of ischaemic heart disease, stroke or peripheral arterial disease.

ACE inhibitor, angiotensin-converting enzyme inhibitor; ARB, angiotensin receptor blocker; HbA1c, glycated haemoglobin.

Trial participants differed substantially from the real-world primary care CKD population. A higher proportion of CREDENCE (100%), DAPA-CKD (67.6%) and EMPA-KIDNEY (44.5%) trial participants had T2D, compared with the real-world CKD population (32.8%). RAS inhibitors were prescribed to almost all trial participants, compared with less than half (45.1%) of the real-world CKD population and under two-thirds (63.3%) of the real-world CKD-T2D population.

### Factors associated with trial eligibility

The logistic regression analyses exploring factors associated with trial eligibility showed that females were less likely than males to be eligible for each trial (Table 4). People of Asian or Black ethnicity were more likely to be eligible for each of the trials than those of White ethnicity. Hypertension was associated with higher odds of being eligible for all three trials, whilst T2D and heart failure were associated with higher odds of being eligible for the DAPA-CKD and EMPA-KIDNEY trials. However, people with heart failure were less likely to be eligible for the CREDENCE trial (OR 0.85, 95% CI 0.776–0.929; *P* < .001). People with CVD were less likely to be eligible for the EMPA-KIDNEY trial (OR 0.90, 95% CI 0.881–0.924; *P* < .001), but were more likely to be eligible for the CREDENCE trial (OR 1.11, 1.042–1.186; *P* < .001). Higher CMMS was associated with greater likelihood of being eligible for the DAPA-CKD (OR 1.15, 95% CI 1.121–1.183; *P* < .001), EMPA-KIDNEY (OR 1.05, 95% CI 1.039–1.071; *P* < .001) and CREDENCE trials (OR 1.12, 95% CI 1.070–1.164; *P* < .001). Use of statins and diuretics were also associated with higher likelihood of being eligible for each of the trials. For the EMPA-KIDNEY trial, the OR for trial eligibility increased with each unit increase in age (OR 1.03, 95% CI 1.031–1.033; *P* < .001), but there was no association for the DAPA-CKD and CREDENCE trials. Individuals within the most deprived category (IMD quintile 1) were more likely to be eligible for the CREDENCE trial than those from the least deprived category (IMD quintile 5) (OR 1.12, 95% CI 1.018–1.239; *P* = .021) but were less likely to be eligible for the EMPA-KIDNEY trial (OR 0.95, 95% CI 0.912–0.979; *P* = .002). People that were underweight were less likely to be eligible for each trial than those of normal weight, however the odds of being eligible for each trial were greater in the higher body mass index categories.

**Table 4:** Multi-variable logistic regression model exploring factors associated with eligibility for each SGLT2 inhibitor kidney outcome trial: primary analysis.

	DAPA-CKD trial					EMPA-KIDNEY trial			CREDENCE trial		
Characteristic	OR		95% CI		*P*-value	OR	95% CI	*P*-value	OR	95% CI	*P*-value
Age (years)	1.00		0.997–1.000		.075	1.03	1.031–1.033	<.001	1.00	0.994–1.000	.054
Gender											
Male	1.00 (Reference)					1.00 (Reference)			1.00 (Reference)		
Female	0.51		0.486–0.527		<.001	0.82	0.806–0.842	<.001	0.56	0.525–0.596	<.001
Ethnicity											
White	1.00 (Reference)					1.00 (Reference)			1.00 (Reference)		
Asian	1.75		1.636–1.869		<.001	1.40	1.336–1.466	<.001	1.87	1.705–2.042	<.001
Black	1.44		1.311–1.571		<.001	1.19	1.117–1.260	<.001	1.41	1.231–1.608	<.001
Mixed	1.53		1.277–1.842		<.001	1.12	0.979–1.272	.100	1.61	1.232–2.093	<.001
Other	1.39		1.140–1.695		.001	1.10	0.960–1.259	.169	1.29	0.957–1.725	.095
IMD quintile											
1 (most deprived)	1.01		0.945–1.071		.852	0.95	0.912–0.979	.002	1.12	1.018–1.239	.021
2	1.02		0.958–1.083		.550	0.99	0.959–1.025	.606	1.12	1.018–1.237	.020
3	1.05		0.989–1.116		.109	1.00	0.970–1.035	.903	1.12	1.016–1.236	.023
4	1.05		0.985–1.112		.138	1.01	0.983–1.047	.372	1.09	0.991–1.209	.075
5 (least deprived)	1.00 (Reference)					1.00 (Reference)			1.00 (Reference)		
Body mass index category											
Underweight	0.72		0.591–0.867		<.001	0.58	0.518–0.651	<.001	0.62	0.430–0.906	.013
Normal weight	1.00 (Reference)					1.00 (Reference)				1.00 (Reference)	
Overweight	1.15		1.088–1.224		<.001	1.23	1.191–1.267	<.001	1.14	1.041–1.249	.005
Obese class I	1.27		1.194–1.351		<.001	1.34	1.294–1.392	<.001	1.27	1.158–1.401	<.001
Obese class II	1.43		1.321–1.542		<.001	1.49	1.430–1.553	<.001	1.43	1.278–1.605	<.001
Obese class III	1.39	1.254–1.542		<.001		1.49	1.409–1.572	<.001	1.43	1.249–1.646	<.001
Comorbidities											
Type 2 diabetes	2.82		2.697–2.948		<.001	1.66	1.626–1.703	<.001			
CVD	1.00		0.957–1.042		.946	0.90	0.881–0.924	<.001	1.11	1.042–1.186	.001
Heart failure	1.07		1.008–1.130		.027	1.51	1.463–1.554	<.001	0.85	0.776–0.929	<.001
Hypertension	3.50		3.253–3.756		<.001	2.61	2.516–2.703	<.001	2.49	2.229–2.772	<.001
CMMS	1.15		1.121–1.183		<.001	1.05	1.039–1.071	<.001	1.12	1.070–1.164	<.001
Medications											
Statin	1.55		1.485–1.627		<.001	1.48	1.449–1.519	<.001	1.96	1.802–2.125	<.001
Diuretic	1.22		1.169–1.275		<.001	1.56	1.525–1.598	<.001	1.19	1.118–1.277	<.001

### Sensitivity analysis

Supplementary data, Fig. S1 illustrates the sensitivity analysis of complete cases for the primary outcome, which identified that 1.3% (*n* = 4740), 3.1% (*n* = 11 516) and 10.4% (*n* = 38 214) of the real-world CKD population (*N* = 367 386) met the eligibility criteria of the CREDENCE, DAPA-CKD and EMPA-KIDNEY trials, respectively. The CREDENCE enrolment criteria applied to 3.0% of the CKD-T2D cohort (*N* = 160 095). The DAPA-CKD enrolment criteria applied to 5.0% (*n* = 8036) of the CKD-T2D cohort and 1.7% (*n* = 3480) of the CKD without T2D cohort, whilst the EMPA-KIDNEY enrolment criteria applied to 13.5% (*n* = 21 579) of the CKD-T2D cohort and 8.0% (*n* = 16 635) of the CKD without T2D cohort. The two sensitivity analyses of complete cases of the logistic regression models exploring factors associated with trial eligibility were generally consistent with the primary analysis (Supplementary data, Tables S4 and S5).

## DISCUSSION

We performed a comprehensive evaluation of the generalizability of three SGLT2 inhibitor kidney outcome trials to a large primary care population with CKD, including those with or without co-existing T2D. We hypothesized that SGLT2 inhibitor kidney outcome trials are more representative of people with co-existing T2D than those without T2D, and that the EMPA-KIDNEY study is more representative than previous trials.

SGLT2 inhibitor kidney outcome trials represent a subgroup of people with CKD who are at high risk of adverse kidney events. In English primary care, <10% of English primary care patients with CKD would have been eligible for each of the SGLT2 inhibitor kidney outcome trials under investigation. The EMPA-KIDNEY trial was the most representative, applying to 8% of the real-world CKD population. This was largely driven by the recruitment of participants without albuminuria, whilst the DAPA-CKD and CREDENCE trials required participants to have significant albuminuria (urine ACR ≥22.6 mg/mmol and >33.9 mg/mmol, respectively). The CREDENCE trial was the least generalizable, applying to only 1% of the real-world CKD population. This was due to the requirement to have both T2D and albuminuria. We also found all three trials to be more representative of patients with CKD and co-existing T2D, compared with those with CKD but without T2D.

Our findings are broadly consistent with previous studies conducted in other settings. Investigators in the USA estimated that between 3% and 10% of people with T2D met trial inclusion criteria, with the lowest proportion eligible for CREDENCE, and the highest proportion eligible for EMPA-KIDNEY [5, 10]. Similar results were reported in a Taiwanese cohort of patients with T2D receiving canagliflozin in the Chang Gung Research Database (*N* = 1479). After applying the trial inclusion criteria, they estimated that only 5% were eligible for the CREDENCE study [6]. A study based in Italy found that 17% of patients with CKD treated in outpatient nephrology clinics, met the DAPA-CKD eligibility criteria [8]. However, this cohort comprised a large proportion of patients with advanced CKD (defined as eGFR <30 mL/min/1.73 m^2^), likely accounting for this notable difference.

In our study, several factors contributed to why people with CKD were ineligible for the trials, including low RAS inhibitor usage and inadequate assessment of albuminuria, which reflect clinician practice rather than trial design. The majority of those ineligible due to not being prescribed a RAS inhibitor either did not meet albuminuria criteria or had not been assessed for albuminuria, reflecting that many people do not have proteinuric kidney disease and that albuminuria is not adequately evaluated. CKD guidelines recommend assessment of urine ACR in people with CKD, and consistent with our findings, it remains poorly implemented in clinical practice, particularly in those without co-existing T2D [21, 22]. Enhanced efforts to test urine ACR in people with CKD are needed to risk stratify and identify those with albuminuria who are most likely to benefit from interventions such as RAS inhibitors and SGLT2 inhibitors. We identified that SGLT2 inhibitor kidney outcome trial participants differed substantially from the real-world English primary care CKD population; trial participants were younger, more likely to have a co-existing T2D and had more advanced kidney disease, with lower eGFR and higher levels of albuminuria, compared with the trial eligible and total CKD primary care populations. In contrast to almost all trial participants, RAS inhibitors were prescribed to less than half of the total primary care CKD population. In addition, females were under-represented in all three SGLT2 inhibitor kidney outcome trials and were less likely than males to be eligible for each trial [23]. These findings were similarly observed in studies evaluating the generalizability of the DAPA-CKD and EMPA-KIDNEY trials to US populations [7, 9].

Understanding how representative trial participants are of a real-world CKD population is important when extrapolating the findings to patients encountered in routine clinical practice [24–26]. Differences in characteristics between trial participants and those receiving the intervention in the real-world may alter its effectiveness and safety profile. It is important to note that the purposeful recruitment of individuals with certain characteristics is valuable in determining treatment effects in subgroups and extending the existing evidence base. The ‘over-representation’ of patients with more advanced kidney disease in the DAPA-CKD and EMPA-KIDNEY trials was intended to determine whether the SGLT2 inhibitor benefits observed in previous trials extended to those with lower eGFR. A lack of representativeness should therefore not necessarily be viewed negatively, but rather as a factor for consideration when applying evidence to patients encountered in routine clinical practice.

Subgroup analyses of SGLT2 inhibitor trials have investigated their effects in different groups of people with CKD. Secondary analysis of the EMPA-KIDNEY study examined the annual rate of decline of kidney function (eGFR slope), demonstrating that the kidney benefits of SGLT2 inhibitors extend to those with lower levels of albuminuria and across a range of eGFRs [27]. The magnitude of effect varied significantly depending on diabetes status and baseline levels of urine ACR and eGFR [27]. However, a recent collaborative meta-analysis of SGLT2 inhibitors showed no significant heterogeneity by diabetes status with regards to kidney outcomes [28]. Clinical trials have not evaluated the kidney efficacy of SGLT2 inhibitors in people with CKD in the absence of RAS inhibition. This remains an important question as many people with CKD are not prescribed a RAS inhibitor. The absence of a RAS inhibitor may not preclude an individual from benefiting from SGLT2 inhibitors, but further data are needed.

These secondary analyses of clinical trials provide valuable insights into the effects of SGLT2 inhibitors in different groups of people with CKD. Real-world evidence can complement this, evaluating the effectiveness of SGLT2 inhibitors in wider, more diverse populations of patients. Importantly, real-world data allow for longer term follow-up in individuals at lower risk of adverse kidney events and in the absence of RAS inhibition. Observational studies utilizing real-world data sources including patient registries, administrative claims and electronic health records are well positioned to facilitate this.

### Limitations

Practices within the Oxford-RCGP RSC network are broadly representative of the English general population but participation in the network is voluntary, resulting in a degree of selection bias [11]. The network has a higher proportion of younger working-aged adults and slightly less deprivation, and practices are unevenly geographically distributed when compared with the national population. The major enrolment criteria were successfully applied to the CKD population, but we were unable to apply some of the minor exclusion criteria, which may have overestimated the number of trial eligible individuals. For example, our finding that the prevalence of CVD in the trial eligible DAPA-CKD cohort was higher compared with the trial cohort could be due to the exclusion criterion for the trial to not have a cardiovascular event within the last 12 weeks before enrolment.

A limitation of identifying trial eligibility criteria from primary care data is missing data and misclassification bias arising from absent or incorrect coding [29]. However, data quality in the Oxford-RCGP RSC network is enhanced by practice engagement through a specialized team of practice liaison officers and ontological mapping to capture data accurately. We identified and adjusted for potential confounders in our models, but unmeasured factors may have resulted in residual confounding, which is a limitation of our multivariable analyses.

## CONCLUSION

SGLT2 inhibitor kidney outcome trials represent a subgroup pf people with CKD that are at high risk of adverse kidney events. In English primary care, <10% of people with CKD would have been eligible for each of the SGLT2 inhibitor kidney outcome trials under investigation. In contrast to trial participants, most people with CKD do not have albuminuria, many do not have co-existing T2D and less than half are prescribed a RAS inhibitor. Our findings highlight the importance of complementing clinical trials with real-world studies, exploring the effectiveness of SGLT2 inhibitors in the broader population of people with CKD treated in routine clinical practice.

## Supplementary Material

gfae071_Supplemental_File

## Data Availability

Access to pseudonymized patient level data will be considered on reasonable request to the corresponding author.
