# Supplementary material for: A comparison of sodium-glucose co-transporter 2 inhibitor kidney outcome trial participants with a real-world chronic kidney disease primary care population
Source: Nephrol Dial Transplant. 2024 Mar 22;40(1):71–82. doi: 10.1093/ndt/gfae071 (PMC11852264; doi:10.1093/ndt/gfae071)
Supplement: gfae071_Supplemental_File [file gfae071_Supplemental_File.docx]

Table S1: SGLT2 inhibitor kidney outcome trial exclusion criteria we were unable to apply to the CKD cohort.

| **DAPA-CKD** |
| --- |
| Immune suppression for kidney disease within 6 months prior to enrolment. |
| Intravenous immune suppression within 3 months prior to enrolment. |
| Myocardial infarction, unstable angina, stroke, or transient ischemic attack within 12 weeks prior to enrolment. |
| Coronary revascularisation or valvular repair/replacement within 12 weeks prior to enrolment or is planned to undergo any of these procedures after randomisation. |
| Active malignancy requiring treatment. |
| Any condition outside the renal and cardiovascular disease area, such as but not limited to malignancy, with a life expectancy of less than 2 years based on investigator´s clinical judgement. |
| Hepatic impairment (aspartate transaminase or alanine transaminase >3x the upper limit of normal, or total bilirubin >2x the upper limit of normal at time of enrolment). |
| Women of child-bearing potential who are not willing to use a medically accepted method of contraception that is considered reliable in the judgment of the investigator OR women who have a positive pregnancy test at enrolment or randomisation OR women who are breast-feeding. |
| Inability of the patient, in the opinion of the investigator, to understand and/or comply with intervention, procedures and/or follow-up OR any conditions that, in the opinion of the investigator, may render the patient unable to complete the study. |
| **EMPA-KIDNEY** |
| Any intravenous immunosuppression therapy in last 3 months; or anyone currently on >45 mg prednisolone. |
| Previous or scheduled bariatric surgery. |
| Ketoacidosis in the past 5 years. |
| Symptomatic hypotension, or systolic blood pressure <90 mmHg or >180 mmHg at screening. |
| Hypersensitivity to empagliflozin or another SGLT2 inhibitor. |
| Medical history that might limit the individual's ability to take trial treatments for the duration of the study. |
| Aspartate transaminase or alanine transaminase >3x the upper limit of normal at screening. |
| Current pregnancy, lactation, or women of childbearing potential, unless using highly effective contraception. |
| Known to be poorly compliant with clinic visits or prescribed medication. |
| **CREDENCE** |
| History of diabetic ketoacidosis. |
| History of hereditary glucose-galactose malabsorption or primary renal glucosuria. |
| Evidence of non-diabetic kidney disease. |
| Renal disease requiring treatment with immunosuppressive therapy. |
| Uncontrolled hypertension. |
| Myocardial infarction, unstable angina, revascularisation procedure, or cerebrovascular accident within 12 weeks before randomisation, or a revascularisation procedure is planned during the trial. |
| Blood potassium >5.5 mmol/mol during screening. |
| Electrocardiogram findings within 12 weeks before randomisation that would require urgent diagnostic evaluation or intervention (e.g., new clinically important arrhythmia or conduction disturbance). |
| Alanine aminotransferase levels >2.0 times the upper limit of normal or total bilirubin >1.5 times the upper limit of normal, unless in the opinion of the investigator and as agreed upon by the sponsor’s medical officer, the findings are consistent with Gilbert’s disease. |
| History of malignancy within 5 years before screening. |
| History of human immunodeficiency virus antibody positive. |
| Major surgery within 12 weeks before randomisation or has not fully recovered from surgery. |
| History of atraumatic amputation within past 12 months of screening, or an active skin ulcer, osteomyelitis, gangrene, or critical ischemia of the lower extremity within 6 months of screening. |
| Use of an aldosterone receptor antagonist or a direct renin inhibitor. |
| Known allergies, hypersensitivity, or intolerance to canagliflozin or its excipients. |
| Pregnant or breast-feeding or planning to become pregnant or breast-feed during the study. |

Table S2: Missing Values for Characteristics of the CKD Cohorts and Trial Eligible CKD Cohorts

|  | Number of Patients with Missing Values (%) | | | | |
| --- | --- | --- | --- | --- | --- |
| Characteristic | **DAPA-CKD Trial Eligible CKD Cohort**  **(N = 11516)** | **EMPA-KIDNEY Trial Eligible CKD Cohort**  **(N = 41209)** | **CREDENCE Trial Eligible CKD-T2D Cohort**  **(N = 4740)** | **CKD Cohort**  **(N = 516491)** | **CKD-T2D Cohort**  **(N = 169443)** |
| Ethnicity | 422 (3.7) | 1868 (4.5) | 177 (3.7) | 27481 (5.3) | 6347 (3.7) |
| Smoking status | 3893 (33.8) | 16210 (39.3) | 1313 (27.7) | 239641 (46.4) | 54319 (32.1) |
| Blood pressure |  |  |  |  |  |
| Systolic | 4394 (38.2) | 16425 (39.9) | 1864 (39.3) | 237861 (46.1) | 72717 (42.9) |
| Diastolic | 4415 (38.3) | 16483 (40.0) | 1870 (39.5) | 238465 (46.2) | 72962 (43.1) |
| BMI | 55 (0.5) | 318 (0.8) | 11 (0.2) | 10381 (2.0) | 654 (0.4) |
| Weight | 39 (0.3) | 209 (0.5) | 11 (0.2) | 9170 (1.8) | 503 (0.3) |
| HbA1c | 37 (0.3) | 140 (0.3) | 0 (0.0) | 1136 (0.2) | 1136 (0.7) |
| eGFR | 0 (0.0) | 0 (0.0) | 0 (0.0) | 22327 (4.3) | 669 (0.4) |
| Urine ACR | 0 (0.0) | 2995 (7.3) | 0 (0.0) | 147466 (28.6) | 9061 (5.3) |

Missing values for HbA1c reported only for patients with diabetes. BMI – body mass index, CKD – chronic kidney disease, CREDENCE — Canagliflozin and Renal Events in Diabetes with Established Nephropathy Clinical Evaluation, DAPA-CKD — Dapagliflozin and Renal Outcomes and Cardiovascular Mortality in Patients with Chronic Kidney Disease, eGFR – estimated glomerular filtration rate, EMPA-KIDNEY — Study of Heart and Kidney Protection with Empagliflozin, HbA1c – glycated haemoglobin, T2D – type 2 diabetes, urine ACR — urine albumin-to-creatinine ratio.

Table S3: Missing Values for Variables Included in Logistic Regression Models of Trial Eligibility

|  | Number of Patients with Missing Values (%) | |
| --- | --- | --- |
| Characteristic | **CKD Cohort (N = 516491)** | **CKD-T2D Cohort (N = 169443)** |
| Ethnicity | 27481 (5.3) | 6347 (3.7) |
| IMD quintile | 22153 (4.3) | 7325 (4.3) |
| BMI | 148878 (28.8) | 17048 (10.1) |

For clinical measures (e.g. BMI) values >2 years from index date classified as missing data. BMI – body mass index, CKD – chronic kidney disease, IMD – index of multiple deprivation, T2D – type 2 diabetes.

Figure S1: Proportion of patients eligible for each SGLT2 inhibitor kidney outcome trial for the total CKD cohort and stratified by type 2 diabetes status; sensitivity analysis using complete cases.

Proportion of CKD population eligible for each of the SGLT2 inhibitor kidney outcome trials for the total CKD cohort and stratified by T2D status. The blue represents the CKD population eligible for the DAPA-CKD study. The red represents the CKD population eligible for the EMPA-KIDNEY study. The green represents the CKD population eligible for the CREDENCE study. N refers to the total number of people in the cohort. CKD — chronic kidney disease, CREDENCE — Canagliflozin and Renal Events in Diabetes with Established Nephropathy Clinical Evaluation, DAPA-CKD — Dapagliflozin and Renal Outcomes and Cardiovascular Mortality in Patients with Chronic Kidney Disease, EMPA-KIDNEY — Study of Heart and Kidney Protection with Empagliflozin, T2D — type 2 diabetes.

Figure S2: Proportion of patients excluded by each eligibility criterion of SGLT2 inhibitor kidney outcome trials.

Exploration of why patients did not meet eligibility criteria of SGLT2 inhibitor kidney outcome trials. The blue represents the DAPA-CKD study, the red represents the EMPA-KIDNEY study, and the green represents the CREDENCE study. Percentages do not add up to 100% as there may be multiple reasons why patients were not trial eligible. CREDENCE —Canagliflozin and Renal Events in Diabetes with Established Nephropathy Clinical Evaluation, DAPA-CKD — Dapagliflozin and Renal Outcomes and Cardiovascular Mortality in Patients with Chronic Kidney Disease, eGFR – estimated glomerular filtration rate, EMPA-KIDNEY — Study of Heart and Kidney Protection with Empagliflozin, HbA1c – glycated haemoglobin, RASi – renin-angiotensin system inhibitor.

Figure S3: Proportion of patients excluded by each exclusion criterion of SGLT2 inhibitor kidney outcome trials.

Exploration of why patients were excluded by each exclusion criterion of the SGLT2 inhibitor kidney outcome trials. The blue represents the DAPA-CKD study, the red represents the EMPA-KIDNEY study, and the green represents the CREDENCE study. Percentages do not add up to 100% as there may be multiple reasons were excluded. AthCVD defined according to EMPA-KIDNEY by the presence of ischaemic heart disease, stroke, or peripheral arterial disease. AAV – antineutrophilic cytoplasmic antibody associated vasculitis, AthCVD – atherosclerotic cardiovascular disease, CCF – congestive cardiac failure, CKD — chronic kidney disease, CREDENCE — Canagliflozin and Renal Events in Diabetes with Established Nephropathy Clinical Evaluation, DAPA-CKD — Dapagliflozin and Renal Outcomes and Cardiovascular Mortality in Patients with Chronic Kidney Disease, eGFR — estimated glomerular filtration rate, EMPA-KIDNEY — Study of Heart and Kidney Protection with Empagliflozin, ESKD – end-stage kidney disease, PKD – polycystic kidney disease, RASi – renin-angiotensin system inhibitor, T1D – type 1 diabetes, T2D – type 2 diabetes.

Table S4: Multi-variable logistic regression model exploring factors associated with eligibility for each SGLT2 inhibitor kidney outcome trial; sensitivity analysis 1.

|  | DAPA-CKD Trial | | | | | EMPA-KIDNEY Trial | | | CREDENCE Trial | | |
| --- | --- | --- | --- | --- | --- | --- | --- | --- | --- | --- | --- |
| Characteristic | **OR** | | **95% CI** | | **p value** | **OR** | **95% CI** | **p value** | **OR** | **95% CI** | **p value** |
| Age (years) | 1.00 | | 0.997 – 1.000 | | 0.124 | 1.03 | 1.030 – 1.033 | <0.001 | 1.00 | 0.994 – 1.000 | 0.084 |
| Gender |  | |  | |  |  |  |  |  |  |  |
| Male | 1.00 [Reference] | | | | | 1.00 [Reference] | | | 1.00 [Reference] | | |
| Female | 0.52 | | 0.493 – 0.539 | | <0.001 | 0.81 | 0.794 – 0.835 | <0.001 | 0.56 | 0.521 – 0.596 | <0.001 |
| Ethnicity |  | |  | |  |  |  |  |  |  |  |
| White | 1.00 [Reference] | | | | | 1.00 [Reference] | | | 1.00 [Reference] | | |
| Asian | 1.64 | | 1.528 – 1.762 | | <0.001 | 1.33 | 1.263 – 1.397 | <0.001 | 1.85 | 1.684 – 2.030 | <0.001 |
| Black | 1.44 | | 1.306 – 1.585 | | <0.001 | 1.22 | 1.141 – 1.300 | <0.001 | 1.41 | 1.222 – 1.612 | <0.001 |
| Mixed | 1.48 | | 1.202 – 1.798 | | <0.001 | 1.12 | 0.963 – 1.286 | 0.137 | 1.50 | 1.116 – 1.973 | 0.005 |
| Other | 1.45 | | 1.173 – 1.783 | | <0.001 | 1.13 | 0.976 – 1.311 | 0.095 | 1.28 | 0.925 – 1.717 | 0.118 |
| IMD Quintile |  | |  | |  |  |  |  |  |  |  |
| 1 (most deprived) | 1.05 | | 0.981 – 1.128 | | 0.153 | 0.98 | 0.942 – 1.021 | 0.350 | 1.15 | 1.035 – 1.277 | 0.009 |
| 2 | 1.06 | | 0.988 – 1.134 | | 0.104 | 1.03 | 0.993 – 1.073 | 0.106 | 1.14 | 1.029 – 1.270 | 0.013 |
| 3 | 1.06 | | 0.993 – 1.137 | | 0.081 | 1.03 | 0.989 – 1.066 | 0.171 | 1.15 | 1.035 – 1.277 | 0.009 |
| 4 | 1.08 | | 1.006 – 1.152 | | 0.032 | 1.05 | 1.010 – 1.087 | 0.013 | 1.13 | 1.017 – 1.258 | 0.023 |
| 5 (least deprived) | 1.00 [Reference] | | | | | 1.00 [Reference] | | | 1.00 [Reference] | | |
| BMI category |  | |  | |  |  |  |  |  |  |  |
| Underweight | 0.96 | | 0.777 – 1.180 | | 0.726 | 0.78 | 0.704 – 0.871 | <0.001 | 0.65 | 0.398 – 1.007 | 0.071 |
| Normal weight | 1.00 [Reference] | | | | | 1.00 [Reference] | | | 1.00 [Reference] | | |
| Overweight | 1.06 | | 1.000 – 1.128 | | 0.051 | 1.14 | 1.107 – 1.183 | <0.001 | 1.13 | 1.026 – 1.245 | 0.013 |
| Obese class I | 1.14 | | 1.065 – 1.213 | | <0.001 | 1.24 | 1.198 – 1.289 | <0.001 | 1.27 | 1.145 – 1.403 | <0.001 |
| Obese class II | 1.26 | | 1.164 – 1.364 | | <0.001 | 1.38 | 1.321 – 1.449 | <0.001 | 1.41 | 1.248 – 1.585 | <0.001 |
| Obese class III | 1.19 | 1.076 – 1.309 | | <0.001 | | 1.36 | 1.285 – 1.444 | <0.001 | 1.43 | 1.241 – 1.642 | <0.001 |
| Comorbidities |  | |  | |  |  |  |  |  |  |  |
| Type 2 diabetes | 2.75 | | 2.617 – 2.895 | | <0.001 | 1.70 | 1.659 – 1.748 | <0.001 | - | - | - |
| CVD | 1.01 | | 0.968 – 1.064 | | 0.548 | 0.89 | 0.867 – 0.915 | <0.001 | 1.13 | 1.057 – 1.214 | <0.001 |
| Heart failure | 1.06 | | 0.998 – 1.132 | | 0.057 | 1.48 | 1.431 – 1.532 | <0.001 | 0.86 | 0.779 – 0.945 | 0.002 |
| Hypertension | 3.40 | | 3.131 – 3.691 | | <0.001 | 2.55 | 2.447 – 2.663 | <0.001 | 2.49 | 2.214 – 2.799 | <0.001 |
| CMMS | 1.15 | | 1.112 – 1.180 | | <0.001 | 1.06 | 1.037 – 1.074 | <0.001 | 1.11 | 1.058 – 1.159 | <0.001 |
| Medications |  | |  | |  |  |  |  |  |  |  |
| Statin | 1.49 | | 1.411 – 1.565 | | <0.001 | 1.46 | 1.422 – 1.503 | <0.001 | 1.85 | 1.691 – 2.020 | <0.001 |
| Diuretic | 1.24 | | 1.178 – 1.297 | | <0.001 | 1.55 | 1.507 – 1.591 | <0.001 | 1.19 | 1.108 – 1.278 | <0.001 |

BMI — body mass index, CI — confidence interval, CMMS — Cambridge multi morbidity score, CREDENCE —Canagliflozin and Renal Events in Diabetes with Established Nephropathy Clinical Evaluation, CVD — cardiovascular disease, DAPA-CKD — Dapagliflozin and Renal Outcomes and Cardiovascular Mortality in Patients with Chronic Kidney Disease, EMPA-KIDNEY — Study of Heart and Kidney Protection with Empagliflozin, IMD — index of multiple deprivation, OR — odds ratio.

Table S5: Multi-variable logistic regression model exploring factors associated with eligibility for each SGLT2 inhibitor kidney outcome trial; sensitivity analysis 2.

|  | DAPA-CKD Trial | | | | | EMPA-KIDNEY Trial | | | CREDENCE Trial | | |
| --- | --- | --- | --- | --- | --- | --- | --- | --- | --- | --- | --- |
| Characteristic | **OR** | | **95% CI** | | **p value** | **OR** | **95% CI** | **p value** | **OR** | **95% CI** | **p value** |
| Age (years) | 1.00 | | 0.997 – 1.002 | | 0.708 | 1.03 | 1.029 – 1.033 | <0.001 | 1.00 | 0.996 – 1.002 | 0.465 |
| Gender |  | |  | |  |  |  |  |  |  |  |
| Male | 1.00 [Reference] | | | | | 1.00 [Reference] | | | 1.00 [Reference] | | |
| Female | 0.54 | | 0.510 – 0.564 | | <0.001 | 0.82 | 0.792 – 0.840 | <0.001 | 0.57 | 0.526 – 0.608 | <0.001 |
| Ethnicity |  | |  | |  |  |  |  |  |  |  |
| White | 1.00 [Reference] | | | | | 1.00 [Reference] | | | 1.00 [Reference] | | |
| Asian | 1.58 | | 1.459 – 1.704 | | <0.001 | 1.33 | 1.254 – 1.404 | <0.001 | 1.76 | 1.592 – 1.941 | <0.001 |
| Black | 1.42 | | 1.280 – 1.581 | | <0.001 | 1.26 | 1.165 – 1.352 | <0.001 | 1.38 | 1.194 – 1.595 | <0.001 |
| Mixed | 1.45 | | 1.152 – 1.802 | | 0.001 | 1.14 | 0.958 – 1.338 | 0.136 | 1.51 | 1.105 – 2.006 | 0.007 |
| Other | 1.42 | | 1.128 – 1.760 | | 0.002 | 1.06 | 0.893 – 1.247 | 0.502 | 1.15 | 0.809 – 1.576 | 0.416 |
| IMD Quintile |  | |  | |  |  |  |  |  |  |  |
| 1 (most deprived) | 1.11 | | 1.031 – 1.203 | | 0.006 | 1.00 | 0.957 – 1.053 | 0.866 | 1.22 | 1.088 – 1.361 | <0.001 |
| 2 | 1.10 | | 1.024 – 1.192 | | 0.010 | 1.04 | 0.994 – 1.090 | 0.085 | 1.20 | 1.074 – 1.343 | 0.001 |
| 3 | 1.11 | | 1.026 – 1.191 | | 0.008 | 1.04 | 0.990 – 1.082 | 0.126 | 1.21 | 1.080 – 1.349 | <0.001 |
| 4 | 1.12 | | 1.040 – 1.208 | | 0.003 | 1.06 | 1.012 – 1.105 | 0.013 | 1.18 | 1.052 – 1.317 | 0.005 |
| 5 (least deprived) | 1.00 [Reference] | | | | | 1.00 [Reference] | | | 1.00 [Reference] | | |
| BMI category |  | |  | |  |  |  |  |  |  |  |
| Underweight | 1.10 | | 0.851 – 1.404 | | 0.444 | 0.81 | 0.694 – 0.941 | 0.007 | 0.82 | 0.486 – 1.301 | 0.437 |
| Normal weight | 1.00 [Reference] | | | | | 1.00 [Reference] | | | 1.00 [Reference] | | |
| Overweight | 1.01 | | 0.941 – 1.075 | | 0.872 | 1.09 | 1.049 – 1.136 | <0.001 | 1.09 | 0.989 – 1.212 | 0.082 |
| Obese class I | 1.09 | | 1.016 – 1.172 | | 0.016 | 1.20 | 1.150 – 1.254 | <0.001 | 1.23 | 1.102 – 1.365 | <0.001 |
| Obese class II | 1.20 | | 1.101 – 1.310 | | <0.001 | 1.33 | 1.257 – 1.403 | <0.001 | 1.35 | 1.192 – 1.535 | <0.001 |
| Obese class III | 1.16 | 1.038 – 1.288 | | 0.008 | | 1.36 | 1.269 – 1.456 | <0.001 | 1.40 | 1.204 – 1.623 | <0.001 |
| Comorbidities |  | |  | |  |  |  |  |  |  |  |
| Type 2 diabetes | 1.88 | | 1.775 – 1.994 | | <0.001 | 1.39 | 1.347 – 1.438 | <0.001 | - | - | - |
| CVD | 1.01 | | 0.959 – 1.063 | | 0.722 | 0.86 | 0.834 – 0.889 | <0.001 | 1.12 | 1.036 – 1.200 | 0.003 |
| Heart failure | 1.08 | | 1.011 – 1.162 | | 0.023 | 1.42 | 1.367 – 1.485 | <0.001 | 0.90 | 0.814 – 1.000 | 0.051 |
| Hypertension | 2.95 | | 2.698 – 3.231 | | <0.001 | 2.27 | 2.160 – 2.390 | <0.001 | 2.54 | 2.241 – 2.887 | <0.001 |
| CMMS | 1.21 | | 1.168 – 1.248 | | <0.001 | 1.13 | 1.111 – 1.159 | <0.001 | 1.14 | 1.085 – 1.195 | <0.001 |
| Medications |  | |  | |  |  |  |  |  |  |  |
| Statins | 1.38 | | 1.305 – 1.465 | | <0.001 | 1.39 | 1.339 – 1.433 | <0.001 | 1.69 | 1.542 – 1.862 | <0.001 |
| Diuretics | 1.21 | | 1.148 – 1.275 | | <0.001 | 1.49 | 1.445 – 1.540 | <0.001 | 1.18 | 1.099 – 1.277 | <0.001 |

BMI — body mass index, CI — confidence interval, CMMS — Cambridge multi morbidity score, CREDENCE —Canagliflozin and Renal Events in Diabetes with Established Nephropathy Clinical Evaluation, CVD — cardiovascular disease, DAPA-CKD — Dapagliflozin and Renal Outcomes and Cardiovascular Mortality in Patients with Chronic Kidney Disease, EMPA-KIDNEY — Study of Heart and Kidney Protection with Empagliflozin, IMD — index of multiple deprivation, OR — odds ratio.

Definition of outlying values

Blood pressure outliers were defined as a systolic blood pressure of <70 mmHg or >260 mmHg and a diastolic blood pressure of <40 mmHg or >150 mmHg or. BMI values were excluded if they were recorded as <10 kg/m^2^ or >100 kg/m^2^. HbA1c outliers were defined as <20 mmol/mol and >200 mmol/mol, and creatinine outliers were defined as <20 µmol/L or >3000 µmol/L.
